# A new oral testosterone (TLANDO) treatment regimen without dose titration requirement for male hypogonadism

**DOI:** 10.1111/andr.13153

**Published:** 2022-01-18

**Authors:** Anthony DelConte, Kongnara Papangkorn, Kilyoung Kim, Benjamin J. Bruno, Nachiappan Chidambaram, Mohit Khera, Irwin Goldstein, Tobias S. Kohler, Martin Miner, Adrian S. Dobs, Mahesh V. Patel

**Affiliations:** ^1^ Lipocine Inc. Salt Lake City Utah USA; ^2^ Pharmaceutical and Healthcare Marketing Saint Joseph's University Philadelphia Pennsylvania USA; ^3^ Department of Urology Baylor College of Medicine Houston Texas USA; ^4^ San Diego Sexual Medicine Alvarado Hospital Medical Center San Diego California USA; ^5^ Department of Urology Mayo Clinic Rochester Minnesota USA; ^6^ Family Medicine and Urology Warran Alpert Medical School Brown University Providence Rhode Island USA; ^7^ Division of Endocrinology Johns Hopkins University Baltimore Maryland USA

**Keywords:** dose titration, efficacy, hypogonadism, oral testosterone, testosterone replacement therapy, testosterone undecanoate

## Abstract

**Background:**

Male hypogonadism (testosterone level < 300 ng/dl) is a clinical syndrome that results from failure of the testis to produce physiological levels of testosterone. Most marketed testosterone replacement therapy products often require multiple dose adjustment clinic visits to achieve the desired, eugonadal testosterone levels.

**Objective:**

To evaluate the efficacy and safety of a novel oral testosterone undecanoate therapy for the treatment of hypogonadism.

**Material and methods:**

Ninety‐five (*N* = 95) hypogonadal men were enrolled in this open‐label, single‐arm, multicenter study in the United States (NCT03242590). Subjects received 225 mg of oral testosterone undecanoate (TLANDO) twice a day for 24 days without dose adjustment. Primary efficacy was percentages of subjects who achieved mean 24‐h testosterone levels within the eugonadal range and secondary efficacies were evaluated based on the upper limit of lab normal range of testosterone concentration.

**Results:**

Subjects enrolled were on average age of 56 years, with about 17% of subjects older than 65 years. The mean body mass index was 32.8 kg/m^2^. The baseline mean total testosterone values were below the normal range (202 ± 74 ng/dl). Post‐treatment with 450 mg testosterone undecanoate daily dose without dose adjustment, 80% of subjects (95% confidence interval of 72%–88%) achieved a testosterone Cavg in the normal range and restored testosterone levels to mean testosterone Cavg of 476 ± 184 ng/dl at steady state. Testosterone restoration was comparable to other approved testosterone replacement therapy products. TLANDO was well tolerated with no deaths, no drug‐related serious adverse events, and no hepatic adverse events.

**Discussion and conclusions:**

TLANDO restored testosterone levels to the normal range in the majority of hypogonadal males. This new oral testosterone replacement therapy can provide an option for no‐titration oral testosterone replacement therapy. This therapy has the potential to improve patient compliance in testosterone replacement therapy.

## 1 INTRODUCTION

Male hypogonadism (testosterone [T] level < 300 ng/dl) is a clinical syndrome that results from failure of the testis to produce physiological levels of T due to disruption of one or more levels of the hypothalamic‐pituitary‐testicular axis.^1^ Primary hypogonadism results from testicular defects, whereas secondary hypogonadism is the failure of the hypothalamus or pituitary gland to produce sufficient gonadotropins. The prevalence of male hypogonadism increases from about 12% for men in their 50s to 50% for men in their 80s.^2^ Furthermore, an estimated 481,000 new cases of T deficiency can be expected per year, in US men, in 40 to 69 years of age.^3^

The Endocrine Society guidelines recommend replacement therapy for symptomatic men with androgen deficiency.^1^ Previously, numerous reports suggested testosterone replacement therapy (TRT) has shown health benefits for bone mineral density, anemia, glycometabolic and cardiometabolic functions, body composition, and improvement in signs and symptoms of mental and sexual functions as well in men with low T levels.^4^, ^5^, ^6^, ^7^ Various TRTs are available in the market including intramuscular injection of T esters, subcutaneous implants, transdermal patches, oral tablets and capsules, buccal, sublingual, nasal, and topical gel formulations of T.

Oral administration of non‐esterified T generally results in low bioavailability as it is extensively metabolized through first‐pass metabolism.^8^ Testosterone undecanoate (TU) is a fatty acid ester of T, with a straight carbon chain (alkylated chain with 11 of carbons) ester at the C17 position of the D‐ring. Oral administration of TU provides a sufficient TU level by lymphatic route absorption through the gastrointestinal tract avoiding the first‐pass metabolism, then TU is converted to T by non‐specific esterases abundant in the body, overcoming the low oral bioavailability of native T.^9^ Oral administration of TU appears to avoid the serious hepatic adverse effects and fatal complications observed after oral administration of 17‐α‐methylated T products.^10^, ^11^ Most marketed TRT products require dose titrations to achieve the eugonadal T levels. One of the top reasons for discontinuation in TRT is lack of perceived efficacy, possibly related to insufficient T levels within the first 3–6 months of therapy, which may be within dose titration duration.^12^

TLANDO is an oral capsule product having 112.5 mg of TU in a unique lipid formulation containing predominantly predigested triglycerides (mono‐ or di‐glycerides). It was designed to enable absorption of TU via the intestinal lymphatic pathway. In a previous 52‐week, multicenter, open‐labeled, active‐controlled study with TLANDO using a dose titration regimen in hypogonadal men (*N* = 315, NCT02081300), it was found that there is little impact of titration for TLANDO on achieving eugonadal total T levels (300–1140 ng/dl).^13^ Titrations in the study were performed two times at weeks 4 and 8 after measuring 24‐h pharmacokinetics (PK) at weeks 3 and 7 to identify who should be titrated, then the final PK measuring at week 13 was performed. As a result, the mean dose for post‐titration was 213 mg TU twice daily (426 mg daily) compared to 225 mg TU twice daily (450 mg daily) for pre‐titration. PK results at week 3 (no titration) showed 86% of subjects within the normal range and the results at week 13 (post two titrations) showed 87% of subjects within the normal range. The distributions of Cavg and Cmax were also shown little impact of titration (*p* = 0.24 for Cavg and *p* = 0.31 for Cmax). With the unique formulation and the previous study PK results on the titration regimen of TLANDO, it was hypothesized that the TLANDO treatment regimen (225 mg BID (twice daily)) may not require dose titration to find an appropriate dose for therapeutic T levels. The goal of this study is to validate the TLANDO dosing regimen without titration and evaluate safety and efficacy.

Here we report the clinical outcomes from this phase III study of oral TU (TLANDO) administered as 225 mg twice daily (450 mg daily) without dose titration.

## 2 MATERIALS AND METHODS

### 2.1 Study design

This was a multicenter, open‐label study evaluating the efficacy of TLANDO with no titration in adult hypogonadal male subjects. The study design and the dose of TLANDO were selected based on results from previous Phase 2 and 3 studies conducted with TLANDO.^13^, ^14^, ^15^ Twelve US‐based study centers participated in this study from January 2017 to April 2017. This study was conducted in accordance with the tenets of the Declaration of Helsinki and complied with International Council for Harmonization and Good Clinical Practice guidelines. The study protocol and informed consent form were reviewed and approved by the single central Institutional Review Board. Written informed consent was obtained from all patients before any study‐related procedures were performed. The study was registered at clinicaltrials.gov under identifier: NCT03242590.

Subjects underwent a screening period to complete the pre‐study examinations and to confirm their hypogonadal status (total T below 300 ng/dl on two consecutive blood samples obtained on separate days at approximately the same time (from 6 to 10 AM each day). Subjects on any T therapy were evaluated for screening following an appropriate washout process. Subjects who met study criteria were enrolled and assigned to receive 225 mg TU twice daily. Each dose was given 12 h apart and administered 30 min after morning and evening meals with no restriction of fat content. No dose adjustment was permitted for the duration of therapy. Following the administration of the morning dose on Day 24 with a meal, intensive PK sampling was carried out for up to 24 h post‐AM dose. Blood samples were obtained at 0 (pre‐dose), 2, 3, 4, 5, 6, 8, and 12 (before evening dose), 14, 15, 16, 17, 18, 20, and 24 h relative to morning dose. A total of 180 ml of whole blood was collected from 15 blood draws per subject. Within 60 min of collection, blood samples were centrifuged for 10 min at ∼1000 x g, and the serum was transferred into cryotubes. Plain red‐top serum tubes (without esterase inhibitor) were used to collect blood samples and the blood was allowed to clot and the serum was removed prior to analysis in this study. Based on a previously performed in vivo phlebotomy study,^16^ there is minimal (∼5%) ex vivo conversion of TU to T during up to 60 min for blood sampling collection process. While this conversion may not be clinically significant, it may have relevance in the evaluation of the T Cmax outliers. Therefore, this was accounted into the Cmax outlier calculation. All serum aliquots were immediately frozen upright at –20°C and maintained frozen until analysis.

### 2.2 Participants

A total of 95 hypogonadal men were enrolled and assigned to receive 225 mg of TLANDO two times a day for about 24 days. Subjects could be naive to T treatment or could enroll after stopping current treatment and completing an adequate washout period. Subjects were diagnosed to be primary (congenital or acquired) or secondary hypogonadal (congenital or acquired) based on symptoms and T levels before enrollment.

Subjects included, otherwise, were healthy men between the ages of 18 and 80 years with a documented history of primary or secondary hypogonadism prior to the age of 65 years. Serum total T below 300 ng/dl was confirmed based on two consecutive morning blood samples, following an appropriate washout of current androgen replacement therapy.

Key exclusion criteria included a history of significant sensitivity or allergy to androgens or product excipients. Also excluded were men with an abnormal prostate as detected through the digital rectal examination. Subjects were also excluded with symptoms of moderate to severe benign prostatic hyperplasia or clinically significant laboratory values including chemistry, hematology, urinalysis, and/or antibodies for hepatitis A, hepatitis B, hepatitis C, or human immunodeficiency virus. Subjects were also excluded based on their medical history including a history of seizure, gastric surgery, cholecystectomy, vagotomy, bowel resection, or any surgical procedure that might interfere with gastrointestinal motility, pH, or absorption.

A complete physical examination was performed at screening and upon study exit and included, at minimum, an examination of head/eyes/ears/nose/throat, breasts, and testis. Heart and lungs were also examined in part of the complete physical examination at screening and exit. The subject's height and weight at screening were used to calculate BMI. Any significant physical examination findings after dosing were recorded as adverse events (AEs).

Blood and urine samples for the clinical laboratory tests were collected for subjects at screening and on Day 24. The test results from screening served as the baseline. Hematology, clinical chemistry, and urinalysis analytes were evaluated. The screening and safety laboratory tests were performed by a central laboratory. Prostate‐specific antigen (PSA), sex hormone‐binding globulin (SHBG), follicle‐stimulating hormone (FSH), luteinizing hormone (LH), hematocrit (HCT), hemoglobin, and prolactin levels were assessed at screening and study exit.

### 2.3 Primary and secondary outcome measures

The definitions of data sets in this study are:

▪ Safety set (SS) includes all subjects who received at least one dose of the study drug (*N* = 95).
▪ Full analysis set (FAS) includes all subjects with at least 1 post‐baseline efficacy variable response (*N* = 94).
▪ Pharmacokinetic set (PKS) includes all subjects in FAS who completed the study without major protocol deviations (*N* = 90).

The primary endpoint was the percentage of TLANDO‐treated subjects who achieved a mean T concentration for 24 h (T Cavg) within the normal range of 300–1080 ng/dl upon completion of 24 days of treatment. The prespecified target responder rate was at least 75%. As prespecified, a 95%, two‐sided, binomial confidence interval (CI) surrounding the point estimate must have a lower bound of 65% or more to conclude that TLANDO treatment is efficacious. The primary endpoint analysis was performed based on the SS population, which included all subjects who received at least one dose of the study drug, with the last observation carried forward including baseline (BLOCF) approach for missing PK data.

The secondary efficacy endpoints were based on the proportion of subjects with the maximum T concentration for 24 h (T Cmax) within target criteria in subjects treated with TLANDO for 24 days. The secondary efficacy endpoint analysis was performed based on the SS as a primary dataset with a model‐based multiple imputation approach to impute missing T Cmax. The target criteria of the secondary efficacy endpoints were:

- T Cmax ≤ 1.5 × the upper limit of normal range (ULN): ≥ 85% of the subjects
- T Cmax between 1.8 × ULN and 2.5 × ULN: ≤ 5% of the subjects
- T Cmax > 2.5 × ULN: no subjects

The serum total T normal range established in this study was from 300 ng/dl to 1080 ng/dl, of which ULN is 1080 ng/dl. Therefore, 1.5 × ULN is 1620 ng/dl, 1.8 × ULN is 1944 ng/dl, and 2.5 × ULN is 2700 ng/dl.

The PKS was used for the PK analysis. Data from subjects with missing concentration values (missed blood draws, lost samples, samples unable to be quantified) were considered as missing and the subject's data were used if the primary PK parameters can be estimated using available data points. Otherwise, those subjects were excluded from the PK analysis.

Serum hormone concentrations were measured during the study confinement period. PK samples were analyzed by a liquid chromatography‐tandem mass spectrometry (LC‐MS/MS) method. Serum concentrations of T, dihydrotestosterone (DHT), TU, dihydrotestosterone undecanoate (DHTU), and estradiol (E2) were determined by using serum extracted from whole blood samples collected at the predetermined times. Each assay method was validated for linearity, precision, accuracy, recovery, and specificity. Analysis of the samples followed the principles of Good Laboratory Practice. In addition to the method validation, the LC‐MS/MS methods were cross‐validated with a Clinical Laboratory Improvement Amendments compliant laboratory (ARUP Labs, Salt Lake City, UT, US) to ensure the normal values can be adopted to the bioanalytical lab.

Safety analysis was carried out throughout the study on all subjects receiving TLANDO and was based on the SS population. Safety was assessed on the basis of AE reports, clinical laboratory data, electrocardiogram (ECG) parameters, physical examinations, and vital sign measurements. Key safety endpoints included incidence of AEs, physical examination results, clinical laboratory test results, and changes in HCT, lipids, serum transaminases, PSA, SHBG, FSH, and LH.

### 2.4 Determination of sample size

The sample size for the study was based on the primary efficacy parameter, the incidence, and binomial CI for the percentage of subjects achieving serum T Cavg within the normal range obtained from previous studies.^14^ In previous safety and efficacy studies, 83%–86% of subjects administered TLANDO 225 mg twice daily had achieved the primary efficacy endpoint (e.g., ≥75% of subjects based on a post hoc analysis). Assuming similar efficacy in the current study, a sample size of 95 subjects would well exceed the 95% CI lower bound of ≥65%.

## 3 RESULTS

### 3.1 Characteristics of the patient population

A total of 95 subjects enrolled in the study and received TLANDO. Ninety‐four (98.9 %) subjects completed the study. One subject was unable to complete the study due to a serious adverse event (SAE) of gastric ulcer hemorrhage, which was not related to the study drug. Overall mean compliance for subjects receiving TLANDO was 99.7 ± 4.9%.

The analysis of baseline characteristics was performed based on the SS population unless otherwise indicated. The demographics, and baseline characteristics, and hormone levels of the patient population are presented in Table 1. The average age of the study population was 56 years of age, with about 17% of subjects being older than 65 years. The race was predominantly White (81%), followed by Black or African American (16%), and others (3%). The mean body mass index (BMI) of subjects was 32.8 kg/m^2^ with 69.5% of subjects having a BMI ≥ 30 kg/m^2^.

**TABLE 1:** Demographics, baseline characteristics, and hormone levels, safety set (*N* = 95)

**Characteristic**	**Value**
Age, years, Mean (SD^†^)	56.0 (8.9)
≤ 65 years, *n* (%)	79 (83.2)
> 65 years, *n* (%)	16 (16.8)
Sex, *n* (%)	
Male	95 (100)
Race, *n* (%)	
Asian	1 (1.1)
Black or African American	15 (15.8)
White	77 (81.1)
Multiple	2 (2.1)
Ethnicity, *n* (%)	
Hispanic or Latino	25 (26.3)
Not Hispanic or Latino	70 (73.7)
Body mass index, kg/m^2^, Mean (SD)	32.8 (5.5)
<25 kg/m^2^, *n* (%)	3 (3.2)
≥25–<30 kg/m^2^, *n* (%)	26 (27.4)
≥30 kg/m^2^, *n* (%)	66 (69.5)
Weight, kg, Mean (SD)	103.6 (18.7)
Baseline T*, ng/dl, Mean (SD)	202 (74.5)
Baseline DHT**, ng/dl, Mean (SD)	18.1 (13.8)
Baseline E2***, pg/ml, Mean (SD)	18.0 (10.2)

^†^ SD: standard deviation.

* Normal range for healthy young adult males for serum total testosterone (T) is 300–1080 ng/dl.

** Normal range for healthy young adult males for serum dihydrotestosterone (DHT) is 10.6–71.0 ng/dl.

*** Normal range for healthy young adult males for serum estradiol (E2) is 10.0–42.0 pg/ml.

All enrolled subjects based on the SS had baseline total T values below the normal range (mean ± SD: 202 ± 74 ng/dl), while 77% had baseline DHT values in the normal or above the normal range (mean ± SD: 18.1 ± 13.8 ng/dl) and 80% had baseline E2 values in the normal or above the normal range (mean ± SD: 18.0 ± 10.2 pg/ml).

### 3.2 Primary and secondary outcome measures

Treatment with TLANDO met the primary efficacy endpoint of the study. 80% of subjects achieved a T Cavg within the normal range at Day 24, which met the pre‐specified target, ≥75%. The lower bound of the 95% CI was 72%, which also met the pre‐specified target, ≥65%. The results for the primary analysis are displayed in Table 2. The additional sensitivity analyses showed that the prespecified targets were not sensitive to subject discontinuations or major protocol violations.

**TABLE 2:** Proportion of subjects achieving T Cavg within the normal range at end of study, safety set (*N* = 95)

**T Cavg responder criteria**	**Proportion of subjects post‐treatment**	
	**Primary efficacy target**	**TLANDO**
Within normal range*	≥ 75%	80%
95% CI (lower, upper bound)**	≥ 65% (lower bound)	72%, 88%

* Serum total testosterone (T) normal range: 300–1080 ng/dl.

** A 95%, 2‐sided, binomial confidence interval surrounding the point estimate was calculated.

The secondary efficacy endpoints were analyzed based on the ULN of serum total T concentration in this study, 1080 ng/dl. The results of the secondary efficacy endpoint analysis are shown in Table 3.

**TABLE 3:** Proportion of subjects achieving T Cmax within the target criteria at end of study, safety set (*N* = 95)

	**Proportion of subjects post‐treatment**	
**T Cmax criteria**	**Secondary efficacy target**	**TLANDO**
≤ 1.5 × ULN*	≥ 85%	82%
Between 1.8 × and 2.5 × ULN*	≤ 5%	5%
>2.5 × ULN*	0%	0%

* ULN is 1080 ng/dl: 1.5 × ULN = 1620 ng/dl, 1.8 × ULN = 1944 ng/dl, and 2.5 × ULN = 2700 ng/dl.

As shown in Table 3, two of three secondary efficacy endpoints were met and the other secondary endpoint was nearly achieved with the TLANDO treatment with no titration.

### 3.3 Pharmacokinetics

**FIGURE 1:**
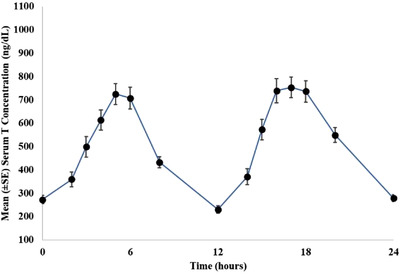
Plot of mean (± standard error [SE]) serum testosterone (T) concentration vs. time after oral testosterone undecanoate (TU) dosing at end of study, pharmacokinetic (PK) set (*N* = 90)

Figure 1 shows the plot of mean serum T concentration with standard errors at each time point versus time after the morning dose at Day 24 (end of study, EOS). As shown in Figure 1, mean serum T concentrations were restored to the normal range within 2 h after TLANDO dose administration. Serum T concentrations reached a peak concentration approximately 4–6 h after dosing. After that, T concentrations declined and approached pre‐dose levels after approximately 12 h post‐administration.

At baseline, the mean T concentration was 202 ng/dl. Mean (±SD) T Cavg obtained from 24‐h PK measurement at EOS was 476 (±174) ng/dl in the PKS (*N* = 90). Mean (±SD) T Cmax measured at EOS in the PKS was 1178 (±484) ng/dl post the AM dose.

At EOS, pre‐dose mean serum T, DHT, TU, DHTU, and E2 concentrations were 270 ng/dl, 88 ng/dl, 13 ng/dl, 12 ng/dl, and 28 pg/ml, respectively. The PK parameters (mean ± SD) for serum T, DHT, TU, DHTU, and E2 after TLANDO administration at EOS in the PK set are shown in Table 4.

**TABLE 4:** Mean pharmacokinetic parameters of testosterone (T), dihydrotestosterone (DHT), testosterone undecanoate (TU), dihydrotestosterone undecanoate (DHTU), and estradiol (E2) in serum after TLANDO dosing at end of study, pharmacokinetic (PK) set (*N* = 90)

**PK parameter**	**T (ng/dl)**	**DHT (ng/dl)**	**TU (ng/dl)**	**DHTU (ng/dl)**	**E2 (pg/ml)**
Pre‐dose (Mean)	270	88	13	12	28
Cavg (Mean ± SD)	476 ± 174	108 ± 46	111 ± 50	47 ± 22	29 ± 13
Cmax (Mean ± SD)	1178 ± 484	179 ± 72	470 ± 256	155 ± 76	44 ± 20
Tmax post‐AM dose, Median h (min, max)	5.0 (1.9, 11.9)	5.9 (1.9, 11.9)	4.9 (1.9, 11.9)	5.0 (1.9, 11.9)	6.0 (2.0, 12.0)

### 3.4 Safety

21.1% (20/95) of subjects experienced at least one treatment‐emergent AE (TEAE). Those TEAEs were mild to moderate, except for one subject with five severe TEAEs; none of these severe TEAEs were considered by the investigator to be related to the study drug. The most frequently reported TEAEs were blood prolactin increase (6.3%), weight increase (2.1%), headache (2.1%), and musculoskeletal pain (2.1%). No deaths were reported during the study. The subject that discontinued experienced an SAE (Gastric Ulcer Hemorrhage), considered not related to study drug but related to other concomitant medications (meloxicam and aspirin). ECG assessments showed abnormal, but not clinically significant findings at baseline for 55 subjects. No clinically significant findings were reported. Mean changes from baseline values in vital sign measurements showed no clinically meaningful changes for heart rate, temperature, or systolic/diastolic blood pressure. During the study, no subject experienced any treatment‐related AEs related to vital sign measurements.

The mean increase in body weight from baseline to study exit was 1.44 ± 3.78 kg. Two subjects (2.1%) had an AE associated with weight increase to mild degree. One of those events was considered by the investigator to be related to the study drug but did not lead to discontinuation. The other subject discontinued the study 3 days after administration of TLANDO.

### 3.5 Hematology, blood chemistry, and urine analysis

HCT values at EOS ranged from 33% to 57% with a mean increase from a baseline of 0.9 ± 3.00%. To minimize the impact of HCT increase, the study had a discontinuation criterion for subjects whose HCT exceeds 54%. None of the subjects met this criterion for discontinuation. One subject who exceeded 54% completed the study and had 57% of HCT at the exit. The subjects (*n* = 5) with T Cmax between 1.8 x ULN and 2.5 x ULN had HCT values within the normal range (40%–52%) at EOS. There were no clinically meaningful changes in hemoglobin values from baseline to EOS (mean change from baseline: 0.02 ± 0.82 g/dl). Hemoglobin values at EOS ranged from 10.7 to 17.1 g/dl.

Mean decreases in lipids were observed (i.e., –1.5 ± 26.2 mg/dl for LDL, –6.9 ± 7.3 mg/dl for HDL, –8.9 ± 85.4 mg/dl for triglycerides, and –10.6 ± 33.1 mg/dl for total cholesterol).

A small increase from baseline for PSA was observed: 0.20 ± 0.44 μg/L (0.80 ± 0.44 μg/L at baseline and 1.00 ± 0.67 μg/L at EOS). There were mean decreases from baseline to EOS for SHBG, LH, and FSH, which were –10.81 ± 7.52 nmol/L, –4.74 ± 4.92 mIU/ml, and –4.91 ± 4.88 mIU/ml, respectively. For FSH and LH, 40% and 41%, respectively, of subjects were maintained within the normal range from baseline to EOS.

## 4 DISCUSSION

There are other commercially available oral TU products. In Gooren's study,^10^ ANDRIOL was administered to 35 hypogonadal patients for a minimum of 10 years. The dose of TU was titrated within 80–200 mg/day (2–5 capsules) and serum T levels resulted in the range between 155 and 187 ng/dl, in which the dose was not appropriate for the therapeutic T levels. However, the patient's sexual functions were improved. The lab test for a 10‐year follow‐up showed no worsening of liver function enzymes. Another oral TU product was recently approved by US Food and Drug Administration (FDA) and requires dose titrations to achieve the therapeutic T levels. A study for the product reported that its starting dose of TU is 237 mg twice daily (474 mg/day) and its post‐titration dose ranges from 158 mg BID (316 mg/day) to 396 mg BID (792 mg/day).^17^ Its plasma T levels were measured using NaF‐EDTA plasma tubes (NaF as esterase inhibitor).

Without dose adjustment, the results of this study demonstrated that the oral twice daily 225 mg TU dose (450 mg daily) of TLANDO is effective in restoring T levels to the normal range in the hypogonadal males. The primary efficacy endpoint was met with 80% of subjects (95% CI lower bound of 72%), achieving serum T Cavg within the normal range at EOS. One subject with missing data was counted as failed. Note that, 20% of subjects who did not achieve T Cavg within the normal range had a mean T Cavg of 245.9 ± 66.4 ng/dl. However, their T levels were increased by 70% from baseline.

It should be noted that the goal of this study was to validate the TLANDO dosing regimen without titration and evaluate safety and efficacy in comparison with the efficacy and safety obtained from the previous 1‐year Phase 3 study of TLANDO with titration.^13^, ^14^ The previous study with titration reported that 86% of subjects achieved T levels within the normal range before titration and 87% were with T levels within the normal range post two titrations.^13^ Therefore, the primary efficacy results in this study confirmed that TLANDO without dose adjustment restores hypogonadal men's T levels to the eugonadal range.

There are pros and cons to using TRT without titration. For example, the absence of dose titrations may eliminate complex titration schemes based on serum T levels, potential titration decision errors, and additional potential clinic/pharmacy visits to seek the right dose for the eugonadal T levels. However, the poor‐ or super‐responders for TRT may not have benefit from a no‐titration regimen. Therefore, criteria for discontinuation for those patients were developed. The criteria require monitoring of serum T concentration (8–9 h after the morning dose) at 3–4 weeks after initiating TLANDO and periodically thereafter. Based on the serum T measurements, the continuation/discontinuation of TLANDO is determined as follows:

- If serum T concentration is within 300–1080 ng/dl: continue TLANDO
- If serum T concentration is <300 ng/dl: discontinue TLANDO
- If serum T concentration is >1080 ng/dl: discontinue TLANDO

Therefore, those patients who do not meet the criteria (<300 ng/dl or >1080 ng/dl) can visit one more time to confirm the levels of T. If T levels are confirmed out of the normal range, then the patients will be discontinued from TLANDO therapy and can pursue another TRT option.

The study drug restored T levels to the eugonadal range in the majority of patients regardless of BMI. Obese subjects (BMI ≥ 30 kg/m^2^, *n* = 65) had 213.4 and 436.9 ng/dl for baseline and EOS, respectively, of mean T Cavg, while non‐obese subjects (BMI < 30 kg/m^2^, *n* = 29) had 177.2 and 557.1 ng/dl (*p* = 0.002), respectively. Two (7%) among non‐obese subjects did not achieve the eugonadal T levels, while 16 (25%) among obese subjects did not reach the eugonadal T levels. This finding may be explained by T distribution into higher tissue mass of obese subjects. Based on quartiles of BMI at baseline, subjects in the 1st quartile (BMI < 28.8 kg/m^2^) had a responder rate of 96%, while the other three quartiles had responder rates of 79%, 71%, and 77% (2nd, 3rd, and 4th quartiles, respectively). This quartile analysis indicates that TLANDO without dose titration is effective in restoring the eugonadal T levels in the majority of patients independently of BMI.

In a separate food effect study with the study drug in 14 hypogonadal males,^15^ mean T Cmax and AUC post‐225 mg TU administration were bioequivalent when TU was administered with food containing low, moderate, or high‐fat amounts. Administration in fasting conditions resulted in approximately 65% and 38% lower Cmax and AUC, respectively, compared to administration with food containing high‐fat amounts. Based on this result, the current study required food but did not limit or restrict the fat amount in meals. As a result, administration of the study drug with food without limitation of fat amounts restored T levels to eugonadal levels in the majority of the hypogonadal men participating in this study.

TLANDO was well tolerated. The occurrence of AEs in this study was consistent with the previous studies.^13^ Overall, 654 hypogonadal subjects were exposed to the study drug in multiple clinical studies including this study. Among those studies, 210 subjects were exposed to the study drug for one year (NCT02081300) while maintaining the T levels.^14^ Their AEs were comparable to the active control (*N* = 105 for ANDROGEL 1.62%) and their quality of life assessed by Psychosexual Daily Questionnaires (PDQ) and 36‐item Short Form survey (SF‐36) was improved compared to the baseline.^18^ In this study, although it was a short study, changes in HCT, PSA, hemoglobin, and lipid levels were consistent with the pharmacologic effects of androgens and similar to those observed with other oral TU products^10^, ^17^ and the previous TLANDO clinical studies.^13^, ^14^, ^15^ No subject had hepatic AEs with TLANDO treatment. The improvement of liver injury markers (alanine aminotransferase, aspartate aminotransferase, alkaline phosphatase, and gamma‐glutamyl transferase) in the majority of subjects was consistent with the results from a clinical study also utilizing the same dosing regimen of oral TU with no titration for four months that measured hepatic fat content.^19^ As an analysis result of secondary efficacy endpoints evaluating T Cmax outliers, two of the three secondary endpoints (1.8 × ULN ≤ T Cmax ≤ 2.5 × ULN in ≤5% of subjects, and T Cmax > 2.5 × ULN in zero subjects) were met (5% and 0%, respectively) while the third secondary endpoint was not met but nearly achieved (82% with T Cmax ≤ 1.5 × ULN, target ≥85%). The results of secondary endpoints were comparable with a recently approved oral TU product with titration (83% with T Cmax ≤ 1.5 × ULN, 3% with 1.8 × ULN ≤ T Cmax ≤ 2.5 × ULN, and 3% with T Cmax > 2.5 × ULN).^17^ This indicates that the risk of excessive T levels is low with a 225 mg BID dose of TLANDO without titration.

Oral administration of TU has been shown to increase ambulatory blood pressures in previous literature.^17^, ^20^, ^21^ The review report of Swerdloff et al. on a recently approved oral TU product with dose titrations showed the oral TU increased mean ambulatory systolic/diastolic blood pressures (BPs) as 4.9 mmHg/2.8 mmHg for 3–4 months in 166 hypogonadal subjects.^17^ Among those subjects, the mean increases in systolic BP were slightly greater in patients with a history of hypertension who were receiving antihypertensive medication (5.5 mmHg) compared to those with no history of hypertension (4.3 mmHg). There were no discontinuations of the study drug due to hypertension. Note that, 5.9% of the oral TU patients were initiated antihypertensive medication or required the dose increase of existing antihypertensive therapy.^17^ Another ambulatory BP study of a developmental oral TU drug with dose titrations reported that the oral TU increased mean ambulatory systolic/diastolic BPs as 1.7 mmHg/0.6 mmHg and 1.8 mmHg/0.6 mmHg for 120 days and 180 days, respectively, in 155 hypogonadal subjects.^20^ The mean increases in systolic BP in patients receiving antihypertensive medication were slightly higher (3.4 mmHg) compared to those without anti‐hypertensive therapy (0.7 mmHg). Note that, 3.2% of the oral TU patients were started on new antihypertensive agents during the 180‐day study.^20^ A recent ambulatory BP study of the current study drug (TLANDO) was performed for four months in 138 hypogonadal subjects.^21^ Note that, 48% of the subjects had a medical history of hypertension or were receiving an anti‐hypertensive medication during therapy. The mean increases of systolic/diastolic BPs post‐4‐month therapy were 3.8 mmHg/1.2 mmHg. Subgroup analysis on hypertensive subjects showed the mean increases in systolic/diastolic BP were slightly higher (4.5 mmHg/1.5 mmHg) compared to subjects without hypertension (3.2 mmHg/0.9 mmHg). Note that, 1% of TLANDO subjects were started on a new anti‐hypertensive medication or required dose increases on the existing anti‐hypertensive medication. There were no deaths and no treatment‐related serious AEs.^21^ The reports of these ambulatory BP monitoring studies with oral TU therapy conclude that although there are slight increases (about 3–5 mmHg) in ambulatory systolic BP with oral TU therapies, the clinical importance of the slight increase in systolic BP in hypogonadal men is unclear since men with T deficiencies have increased in cardiovascular risk.^22^

In the current study, a clinical cuff BP measure was performed at baseline and each visit during the study. The oral TU did not increase clinical cuff systolic BP (–0.5 ± 13.53 mmHg) and diastolic BP (–1.0 ± 7.95 mmHg) at 24 days in 94 hypogonadal subjects. There were no clinically meaningful BP changes.

In this study, 17% of enrolled subjects were greater than 65 years of age. There were no geriatric subjects with drug‐related AEs during treatment. A post hoc analysis showed the geriatric subpopulation had mean levels of T Cavg as 513.5 ± 233.8 ng/dl while the subpopulation ≤ 65 years had 466.5 ± 170.0 ng/dl (*p* = 0.41), respectively. Although a small population, this geriatric subpopulation analysis may support the use of TLANDO in the geriatric population (over 65 years of age).

## 5 CONCLUSIONS

A 225 mg BID oral dose of TLANDO (450 mg total daily dose) restored serum T levels to the normal range without the need for dose adjustment in hypogonadal men. TLANDO offers an alternative option for prescribers and patients with primary or secondary hypogonadism. TLANDO therapy without dose titration may improve patient compliance.

## CONFLICT OF INTEREST

Anthony DelConte is a paid consultant to Lipocine. Kongnara Papangkorn, Kilyoung Kim, Benjamin J Bruno, Nachiappan Chidambaram, and Mahesh V Patel are employees of Lipocine. Mohit Khera, Irwin Goldstein, Tobias S Kohler, Martin Miner, and Adrian S Dobs are consultants to Lipocine and have no commercial relationship with Lipocine.

## AUTHOR CONTRIBUTIONS

Anthony DelConte, Nachiappan Chidambaram, and Mahesh V Patel designed the study, interpreted the results, and edited the manuscript. Kongnara Papangkorn and Kilyoung Kim analyzed the data, interpreted the results, and drafted the manuscript. Benjamin J Bruno, Mohit Khera, Irwin Goldstein, Tobias S Kohler, Martin Miner, and Adrian S Dobs interpreted the results, revised and edited the manuscript.
